# 16S rRNA Gene Amplicon Sequence Data from Feces of Wild Deer (Cervus nippon) in Japan

**DOI:** 10.1128/MRA.00346-20

**Published:** 2020-05-28

**Authors:** Kasumi Ishida-Kuroki, Nachiko Takeshita, Yoshihiro Nitta, Takehisa Chuma, Ken Maeda, Hiroshi Shimoda, Ai Takano, Tsutomu Sekizaki

**Affiliations:** a Research Center for Food Safety, Graduate School of Agricultural and Life Sciences, The University of Tokyo, Tokyo, Japan; b Laboratory of Veterinary Public Health, Joint Faculty of Veterinary Medicine, Kagoshima University, Kagoshima, Japan; c Laboratory of Veterinary Microbiology, Joint Faculty of Veterinary Medicine, Yamaguchi University, Yamaguchi, Japan; d Laboratory of Veterinary Epidemiology, Joint Faculty of Veterinary Medicine, Yamaguchi University, Yamaguchi, Japan; Indiana University, Bloomington

## Abstract

We report 16S rRNA amplicon sequence data from feces of 109 wild deer in Japan. The dominant bacterial taxa in fecal microbiota of wild deer hunted between village and mountainous areas and those living on Miyajima Island and in Nara Park were similar but differed in abundance.

## ANNOUNCEMENT

Gut microbiota of humans and animals are important for health, nutrition, and physiology and are affected by host diet and phylogeny (1, 2). However, information on the microbiota of wild animals is still limited (3). In this study, we collected feces of wild deer in several areas of Japan and analyzed the microbiota.

Rectal feces were collected from 34 wild deer (Cervus nippon nippon) in Yamaguchi Prefecture, Japan, and 25 wild deer (*Cervus nippon* *nippon*) in Kagoshima Prefecture, Japan. These deer were killed by licensed hunters between village and mountainous areas between 2014 and 2019. The rectum was aseptically removed from the carcass, and the feces were collected in sterilized tubes. Furthermore, dropped feces of 50 wild deer (*C. nippon nippon* and *C. nippon aplodontus*) were collected in Miyajima Island and Nara Park, Japan, respectively. The feces were quickly picked up with rubber gloves after being dropped on the ground and were collected in sterilized tubes. Miyajima Island is a major tourist spot in Hiroshima Prefecture, and several deer are seen around the ferry port and shrine; however, feeding the deer is prohibited, and most deer inhabit the mountainous part of the island. Nara Park is also a tourist spot, and tourists can feed the deer; therefore, many deer come closer to and even touch people. All samples were immediately cooled and stored at −20°C until use. Frozen samples were thawed and centrifuged at 13,000 × *g* for 5 min at 4°C. The pellet was washed twice with sterile 0.85% saline, and DNA was extracted as described previously (4).

The V3-V4 regions of 16S rRNA genes in the extracted DNA were amplified with primers 341F (5′-ACACTCTTTCCCTACACGACGCTCTTCCGATCT-NNNNN-CCTACGGGNGGCWGCAG-3′) and 805R (5′-GTGACTGGAGTTCAGACGTGTGCTCTTCCGATCT-NNNNN-GACTACHVGGGTATCTAATCC-3′) (5), including an overhang adapter sequence (Illumina, San Diego, CA, USA). Sequencing was performed using the 2 × 300-bp paired-end method on the MiSeq platform with a MiSeq v3 reagent kit (Illumina). FASTQ reads were processed using the IM-TORNADO pipeline v2.0.3.2 (6) with default parameters, except for Trimmomatic (LEADING:20, TRAILING:20, and MINLEN:180). The reads were filtered for quality using Trimmomatic and merged using scripts in the pipeline. A total of 12,419 to 31,939 high-quality reads were obtained from each sample. The pipeline used mothur (7) for operational taxonomic unit (OTU) clustering at 100% sequence identity and a k-mer-based approach for taxonomy assignment using the Ribosomal Database Project (RDP) naive Bayesian classifier (8) with a threshold of 80% bootstrap confidence. Each OTU was assigned at the family level against the RDP database (9) at 97% sequence identity. The number of sequences was normalized to 12,419 for each sample by random subsampling and was used for a nonmetric multidimensional scaling (NMDS) based on Bray-Curtis dissimilarity. The NMDS plot was generated using the vegan package in R (10). The study was confirmed to not require approval under the code of ethics for animal experiments by the Animal Research Committee of The University of Tokyo.

**FIG 1:**
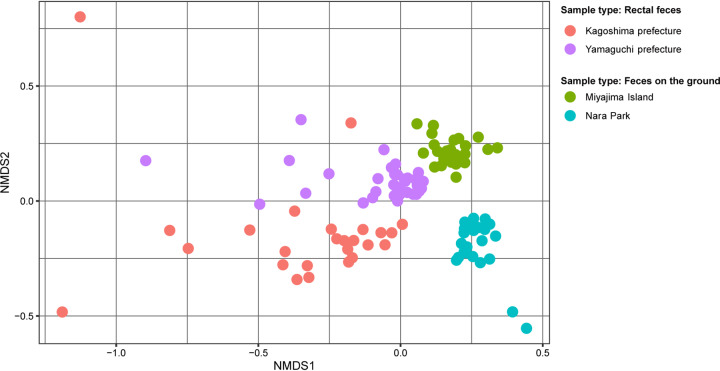
Nonmetric multidimensional scaling (NMDS) plot based on Bray-Curtis distances. Samples are colored by sampling location.

Taxonomic classification at the family level showed that the bacterial taxa were similar among the four groups but differed in abundance. *Ruminococcaceae* (16.2 to 29.7%) was the most abundant taxon in all groups. *Lachnospiraceae* was second in deer from Yamaguchi Prefecture (12.8%) and Kagoshima Prefecture (15.1%) but third in deer from Miyajima Island (7.8%) and fourth in those from Nara Park (7.0%). The second most abundant taxa in deer from Miyajima Island and Nara Park were *Peptostreptococcaceae* (12.4%) and *Bacteroidaceae* (12.7%), respectively. An NMDS plot shows that samples formed clusters by sampling region (Fig. 1).

### Data availability.

The data sets generated from 16S rRNA gene amplicon sequencing in this study have been deposited in the NCBI Sequence Read Archive (SRA) under accession numbers DRA009920, DRA009921, DRA009922, and DRA009923 for Yamaguchi Prefecture, Kagoshima Prefecture, Miyajima Island, and Nara Park, respectively.
